# Heme-Dependent ER Stress Apoptosis: A Mechanism for the Selective Toxicity of the Dihydroartemisinin, NSC735847, in Colorectal Cancer Cells

**DOI:** 10.3389/fonc.2020.00965

**Published:** 2020-06-17

**Authors:** Ahmed E. M. Elhassanny, Eman Soliman, Mona Marie, Paul McGuire, Waseem Gul, Mahmoud ElSohly, Rukiyah Van Dross

**Affiliations:** ^1^Department of Pharmacology and Toxicology, Brody School of Medicine, East Carolina University, Greenville, NC, United States; ^2^Department of Pharmacology and Toxicology, Faculty of Pharmacy, Zagazig University, Zagazig, Egypt; ^3^Division of Hematology/Oncology, Department of Internal Medicine, Brody School of Medicine, East Carolina University, Greenville, NC, United States; ^4^Medical Doctor Program, Brody School of Medicine, Greenville, NC, United States; ^5^ElSohly Laboratories Inc., Oxford, MS, United States; ^6^National Center for Natural Products Research, School of Pharmacy, The University of Mississippi, Oxford, MS, United States; ^7^Center for Health Disparities, East Carolina University, Greenville, NC, United States

**Keywords:** artemisinin, colorectal cancer, selective toxicity, endoplasmic reticulum stress, reactive oxygen species, iron, heme, FOLFOX

## Abstract

Colorectal cancer (CRC) is a leading cause of cancer death in the United States. Artemisinin derivatives, including the dihydroartemisinin (DHA) monomers, are widely used as clinical agents for the treatment of malaria. Numerous studies demonstrate that these molecules also display antineoplastic activity with minimal toxicity. Of interest, dimeric DHA molecules are more active than their monomeric counterparts. Our previous data showed that the DHA dimer, NSC735847, was a potent inducer of death in different cancer cell types. However, the mechanism of action and activity of NSC735847 in colon cancer cells was not explored. The present study investigated the anticancer activity of NSC735847 and four structurally similar analog in human tumorigenic (HT-29 and HCT-116) and non-tumorigenic (FHC) colon cell lines. NSC735847 was more cytotoxic toward tumorigenic than non-tumorigenic colonocytes. In addition, NSC735847 exhibited greater cytotoxicity and tumor selectivity than the NSC735847 derivatives. To gain insight into mechanisms of NSC735847 activity, the requirement for endoplasmic reticulum (ER) stress and oxidative stress was tested. The data show that ER stress played a key role in the cytotoxicity of NSC735847 while oxidative stress had little impact on cell fate. In addition, it was observed that the cytotoxic activity of NSC735847 required the presence of heme, but not iron. The activity of NSC735847 was then compared to clinically utilized CRC therapeutics. NSC735847 was cytotoxic toward colon tumor cells at lower concentrations than oxaliplatin (OX). In addition, cell death was achieved at lower concentrations in colon cancer cells that were co-treated with folinic acid (Fol), 5-FU (F), and NSC735847 (FolFNSC), than Fol, F, and OX (FolFOX). The selective activity of NSC735847 and its ability to induce cytotoxicity at low concentrations suggest that NSC735847 may be an alternative for oxaliplatin in the FolFOX regimen for patients who are unable to tolerate its adverse effects.

## Introduction

Colorectal cancer (CRC) is the third most frequently diagnosed cancer in the United States (1). CRC is also the third leading cause of cancer-related deaths in women and the second leading cause in men with 51,000 deaths estimated for 2019 (2). CRC is managed according to patient and tumor characteristics (3, 4). In general, non-metastatic CRC is treated by surgically resecting the lesion. In metastatic CRC, surgery is accompanied with the administration of cytotoxic, targeted, or immunomodulatory chemotherapeutic agents (1). In the advanced CRC setting, the standard of care chemotherapeutic regimen consists of a combination of folinic acid (Fol), 5-fluorouracil (F or 5-FU) and oxaliplatin (OX), also referred to as FolFOX. This regimen provides significant clinical benefit for CRC however, OX produces serious, grade 3 neurotoxicities that can lead to the discontinuation of treatment and a substantial reduction in the patient's quality of life (5, 6). OX is an alkylating agent that induces severe DNA damage in cancer cells. Unfortunately, OX is not selectively toxic toward cancer because it also damages DNA in the peripheral nerves (7). Therefore, agents that selectively target colon cancer cells while heightening the antitumor activity of the Fol plus F combination, are sorely needed for patients who are unable to tolerate the adverse effects of FolFOX.

Artemisinin is a naturally occurring, endoperoxide-containing sesquiterpene lactone. This agent and its derivatives, including dihydroartemisinin (DHA), are used throughout the world as first-line treatments for malaria (8, 9). In addition to their impressive anti-malarial activity, artemisinins produce few toxicities and thus have an excellent safety profile (10, 11). Of importance, artemisinin and its derivatives also display activity against malignancies including prostate, pancreatic, and colon cancer (12, 13). Both monomeric and dimeric artemisinin molecules are efficacious against cancer, with the dimers displaying better stability and greater activity than the monomers (14). The antineoplastic activity of artemisinin monomers and dimers has been attributed to the activation of their endoperoxide moiety to free radicals that generate reactive oxygen species (ROS) (15). Low to moderate levels of ROS promotes survival by stimulating cellular processes, including proliferation and differentiation, that permit greater endurance against cellular stressors. However, excessive ROS production causes death by damaging cellular components including DNA, proteins, and lipids (16). Interestingly, cancer cells have higher levels of ROS than normal cells. Therefore, it has been proposed that pharmacological ROS inducers elicit selective toxicity by causing ROS levels to exceed the cytotoxic threshold in cancer but not in non-cancer cells that have low endogenous ROS levels (16, 17).

The cytotoxicity of artemisinin-type molecules has also been linked to the endoplasmic reticulum (ER) stress pathway (18–21). ER stress occurs when the load of unfolded proteins exceeds the protein folding capacity of the cell (22). In response to ER stress, unfolded substrates cause GRP78/BiP to release three ER stress sensors; double-stranded RNA-activated protein kinase (PRK)-like endoplasmic reticulum kinase (PERK), activated transcription factor 6 (ATF6), and inositol requiring kinase-1 (IRE1α). Activation of these ER stress sensors blocks the translation of proteins, enhances the expression of protein chaperones, and increases the degradation of ER-resident proteins to resolve the stress (23). However, severe or overwhelming ER stress upregulates the expression of proapoptotic proteins including the transcription factor, C/EPB homologous protein10 (CHOP10) (24). Cancer cells contain low to moderate levels of ER stress primarily due to the increased demand for folded proteins that are utilized for cell cycle progression and survival (25). Similar to ROS inducers, ER stress inducing agents exploit the difference in ER stress levels between tumor and non-tumor cells to confer selective toxicity (26). Therefore, it is of interest that clinical and investigational agents of diverse chemical structure, including doxorubicin, bortezomib, and artemisinin derivatives, elicit ER stress as primary or secondary modes of drug action (27, 28).

Previous studies from our group demonstrated that the DHA dimer, NSC735847, was cytotoxic against different cancer cell lines *in vitro* and *in vivo* (18, 29). We found that NSC735847 was a potent inducer of ROS and that iron and heme promoted ROS-induced cell death in the promyelocytic leukemia cell line, HL-60 and the prostate cancer cell line, PC3. In addition, NSC735847 increased the expression of ER stress-related proteins. However, the mechanism of NSC735847 cytotoxicity in CRC and its selectivity toward cancer have not been explored. Therefore, the current study examined the antitumor activity of NSC735847 and its structural analogs to identify lead compounds that were efficacious and selectively active against CRC cells. Our primary goal was to define the mechanism of action of the lead compound to guide the selection of FDA approved, CRC antineoplastic agents with which it could be co-administered to enhance the overall antitumor response.

## Materials and Methods

### Antibodies and Reagents

Folinic acid, Trolox, salubrinal, succinylacetone, and β-actin antibody were purchased from Sigma-Aldrich (St. Louis, MO). Fluorouracil was from LKT Laboratories (St. Paul, MN). Oxaliplatin was from LC Laboratories (Woburn, MA). Antibodies directed toward full-length (FL)/cleaved caspase-3, FL/cleaved PARP, phospho-eIF2α (P-eIF2α), total eIF2α (T-eIF2α), P-PERK, and total-PERK were from Cell Signaling Technology (Beverly, MA). Anti-CHOP10 antibody was from Santa Cruz Biotechnology (Santa Cruz, CA). Anti-GAPDH antibody and GSK2606414 were obtained from EMD Millipore (Burlington, MA). Anti-ferritin antibody was purchased from Abcam (Cambridge, MA). Anti-rabbit 800CW and anti-mouse 680RD secondary antibody IRDyes were from LI-COR Biosciences (Lincoln, NE). The heme oxygenase inhibitor, QC-308, was purchased from AsisChem Inc. (Waltham, MA).

### Cell Culture

The human colon cancer cell lines HT29 and HCT116 were cultured in McCoy's 5A medium (Sigma Aldrich, St. Louis, MO) containing 10% heat-inactivated fetal bovine serum (FBS), penicillin (100 units/ml), and streptomycin (100 μg/ml). The non-tumorigenic colon cell line, FHC, was cultured in DMEM: F12 (1:1) medium supplemented with 10% heat inactivated FBS, 25 mM HEPES (Thermo Fisher Scientific Inc., IL), 10 ng/ml cholera toxin (Sigma Aldrich, St. Louis, MO), 0.005 mg/ml insulin (Thermo Fisher Scientific Inc., IL), 0.005 mg/ml, transferrin (Sigma Aldrich, St. Louis, MO), 100 ng/ml hydrocortisone (Sigma Aldrich, St. Louis, MO), 20 ng/mL human recombinant epidermal growth factor (Thermo Fisher Scientific Inc., IL), 100 units/ml penicillin, and 100 μg/ml streptomycin.

### MTS Cell Viability Assays

Cells were cultured in 96-well-plates for 48 h before treatment. Serum-free media containing different agents was added to the cells at the concentration and time period described in the figure legend. MTS reagent (Promega, Madison, WI) was then added to each well and the absorbance was measured at 495 nm as directed by the manufacturer. In the presence of MTS reagent, the absorbance reading is proportional to the number of viable cells. The half-maximal inhibitory concentration (IC_50_) of the tested compounds is the concentration that reduces the viability of cells by 50%. IC_50_ was calculated after 24 h of treatment (30, 31) as described previously (32–34) from dose-response curves (log drug concentration vs. percentage viability from untreated cells) generated by non-linear regression analysis with GraphPad Prism 5 software (GraphPad Software, San Diego, California).

### Caspase 3/7 Activity Assay

The cells were plated in white-walled 96-well-plates and then they were cultured at 37°C for 48 h. Serum-free culture medium containing the appropriate concentration of different agents was added to the cells for the indicated period of time. Caspase-Glo 3/7 reagent (Promega, Madison, WI) was added to each well and luminescence was detected as directed by the manufacturer. The Caspase-Glo 3/7 kit measures the activity of the executioner caspases 3 and 7 using the luminogenic substrate (Z-DEVD-aminoluciferin) as substrate.

### TUNEL Assay

HT29, HCT116, and FHC cells were cultured in chamber slides and treated with 5 μM NSC735847, 10 μM NSC735847, or vehicle (0.1% DMSO). The cells were then washed with PBS and fixed in 4% paraformaldehyde before detection of double stranded DNA breaks that are characteristic of apoptosis. Terminal deoxynucleotidyl transferase dUTP nick end labeling (TUNEL) assays were performed as described by the manufacturer (*in situ* Cell Death Detection Kit; Roche; Indianapolis, IN). Briefly, fixed cells were incubated in permeabilization solution (0.1% Triton X-100 in 0.1% sodium citrate) for 2 min on ice. The slides were rinsed twice with PBS, 50 μL TUNEL reaction mixture was added, and then the samples were incubated for 1 h at 37°C in the dark. The slides were overlayed with antifade mounting medium containing DAPI and then visualized using a Zeiss LSM 700 confocal microscope system (Carl Zeiss Microscopy, LLC, White Plains, NY).

### Western Blot Analysis

Western blot analysis was conducted as described previously (35). Briefly, the cells were cultured in 100 mm dishes for 48 h before treatment. The cells were then treated as described in the figure legends. For vehicle-treated samples, DMSO was added to serum-free culture medium at a maximum concentration of 0.1% (v/v). After experimentation, the cells were washed twice with phosphate buffer saline (PBS) and then 100 μl of Triton lysis buffer [25 mM HEPES, 100 mM NaCl, 1 mM EDTA, 10% (v/v) glycerol, 1% (v/v) Triton X-100], containing protease and phosphates inhibitors, was added to the cells. Protein concentrations were determined by using BCA reagents (Thermo Fisher Scientific Inc., IL). Equal concentrations of each sample were loaded onto SDS–PAGE gels and protein bands transferred to nitrocellulose membranes using semi-dry transfer cells (TRANS-BLOTSD; Bio-Rad Laboratories, Hercules, CA). The membranes were incubated for 2 h at room temperature in Odyssey blocking buffer (LI-COR Biosciences, Lincoln, NE). Membranes were then incubated overnight at 4C with primary antibody followed by 1 h of incubation at room temperature with secondary antibody. All primary antibodies were used at a dilution of 1:1,000 except anti-GAPDH and anti-CHOP10 antibodies which were used at 1:10,000 and 1:500 dilutions, respectively. All secondary antibodies were used at a dilution of 1:15,000. Protein bands were visualized using Odyssey® CLx digital fluorescence imaging system (LI-COR, Lincoln, Nebraska). Band intensities were quantified by using ImageJ software (36).

### Oxidative Stress Measurement

Oxidative stress was measured in cultured cells by using the probe, chloromethyl-2′,7′ dichlorodihydrofluorescein diacetate (CM-H_2_DCFDA or DCF) purchased from Life Technologies (Grand Island, NY). DCF is a chemically reduced form of fluorescein used as an indicator for reactive oxygen species (ROS) in cells. The cells were loaded with 5 μM DCF in phenol red-free, serum-free medium for 30 min and then the cells were treated with the appropriate drug for the indicated period of time. The cells were then trypsinized and resuspended in serum-containing, phenol red-free medium. The fluorescence of DCF was measured using an Accuri C6 flow cytometer (BD Accuri Cytometers, Ann Arbor, MI) at an excitation wavelength of 488 and emission of 533 ± 30 nm.

### Quantitative Reverse Transcriptase-Polymerase Chain Reaction (qRT-PCR)

Total RNA was extracted from HT29 cells by utilizing TRI reagent (Sigma Aldrich, St. Louis, MO, USA) and then it was purified with the Direct-zol RNA MiniPrep Kit (Zymo Research, Irvine, CA, USA). cDNA was synthesized from purified RNA by using the iScript cDNA Synthesis Kit (Bio-Rad laboratories, Hercules, CA, USA). qRT-PCR was performed by using the iTaq Universal SYBR Green Supermix (Bio-Rad laboratories, Hercules, CA, USA) with the BioRad CFX96 thermocycler (Bio-Rad laboratories, Hercules, CA, USA). The specific primer pair sequences were 5′-CTACGTCGCCCTGGACTTCGAGC-3′ and 5′-GATGGAGCCGCCGATCCACACGG-3′ for β-actin; and 5′-CCCCCATTTGTGTGACTTCAT-3′ and 5′-GCCCGAGGCTTAGCTTTCATT-3′ for ferritin H (heavy-chain). Cycle threshold values were normalized to the internal beta-actin control. The ratio of fold change was calculated using the Pfaffl method (37).

### Intracellular Iron Measurements

HT29 cells were plated in 100 mm tissue culture plates for 48 h and then the cells were treated as indicated in the text. The cells were washed twice with PBS and then 100 μl triton lysis buffer (without EDTA) was added. The intracellular iron concentrations were determined using the QuantiChrom iron assay kit (BioAssay Systems; #ab83366) following the manufacturer's instructions. Briefly, iron standards of different concentrations were prepared by diluting the iron standard provided with the kit with distilled water. The diluted standards (50 μL) and sample cell lysates (50 μL) were transferred into a clear flat bottom 96-well-plate. Working reagent was prepared by mixing 20 volumes of reagent A, 1 volume reagent B and 1 volume reagent C provided with the kit. The working reagent (200 μL) was then added to the standard and sample wells. After mixing, the plate was incubated 40 min at room temperature and then the absorbance was measured at 590 nm. The iron concentration in each sample was calculated as described in the kit protocol.

### Statistical Analysis

All data are representative of three or more independent experiments. Data are presented as the mean ± standard error of mean (SEM). One-way analysis of variance (ANOVA) followed by Tukey's *post-hoc* analysis was carried out using GraphPad Prism 5 software (GraphPad Software, San Diego, California). With regard to comparisons, values were considered significant at *p* < 0.05.

## Results

### NSC735847 Is Selectively Toxic Toward Colon Cancer Cells

**Figure 1:**
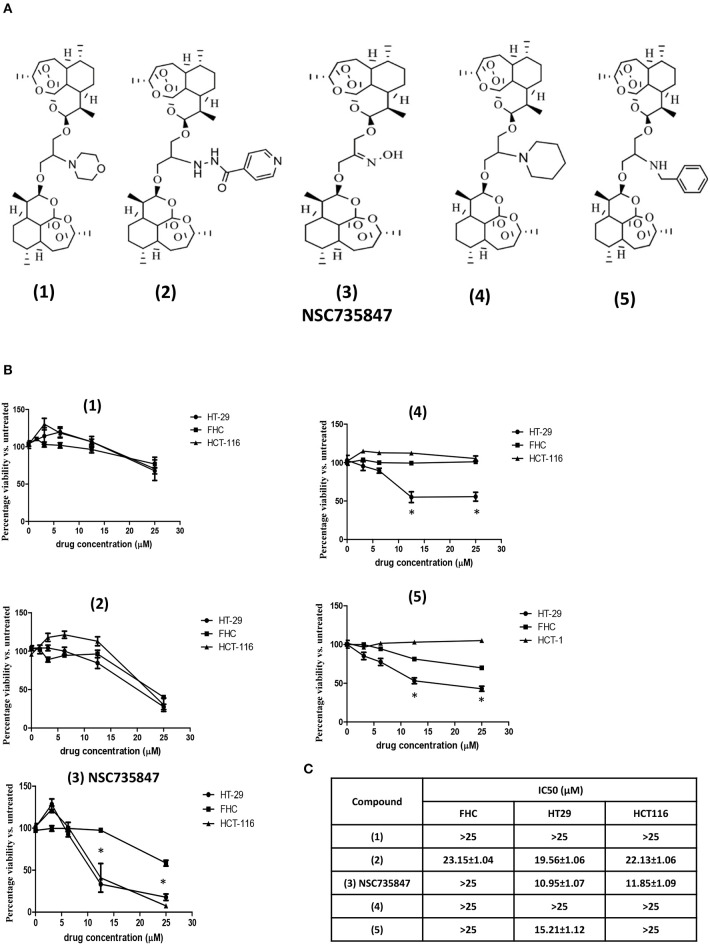
Dihydroartemisinin dimer structures and their cytotoxic activities against colorectal cancer and non-tumorigenic colon cells. **(A)** Chemical structures of the dihydroartemisinin dimers used in this study **(B,C)** Effect of dihydroartemisinin dimers on the viability of human colon cancer cells (HT29 and HCT-116) and non-tumorigenic colon cells (FHC). **(B)** Cell survival was measured by conducting MTS viability assays after 24 h of treatment with vehicle (0.1% DMSO) or the dihydroartemisinin dimers at doses of 3.1, 6.25, 12.5, and 25 μM. **(C)** IC_50_ values (the drug concentration that reduced the viability of cells by 50%) were calculated using non-linear regression analysis of GraphPad Prism 5 software. Data are represented as the mean ± SEM of three independent experiments (*indicates a statistically significant difference between tumor and non-tumor cells, *P* < 0.05).

Our previous studies showed that the DHA dimer, NSC735847, inhibited the survival of leukemia (HL-60) and prostate cancer (PC-3) cell lines (18). In the current study, we sought to determine if NSC735847 or structurally similar analogs (Figure 1A), were effective and selectively toxic toward colon cancer. Selectively toxic agents were defined as those that elicited significantly greater cytotoxicity toward human tumorigenic (HT-29 and HCT-116) than non-tumorigenic (FHC) colonocytes after 24 h of treatment (Figure 1B). In cells treated with compound **(3)** (NSC735847), the survival of HT-29 and HCT-116 cancer cells (IC_50_ = 10.95 and 11.85 μM, respectively) was significantly reduced compared to non-cancerous, FHC colonocytes (IC_50_ > 25 μM) (Figures 1B,C). Compounds **(4)** and **(5)** were more cytotoxic toward HT-29 than FHC cells, however these derivatives were inactive against HCT-116 cells. In contrast, compounds **(1)** and **(2)** were not selectively cytotoxic toward either colon cancer cell line.

**Figure 2:**
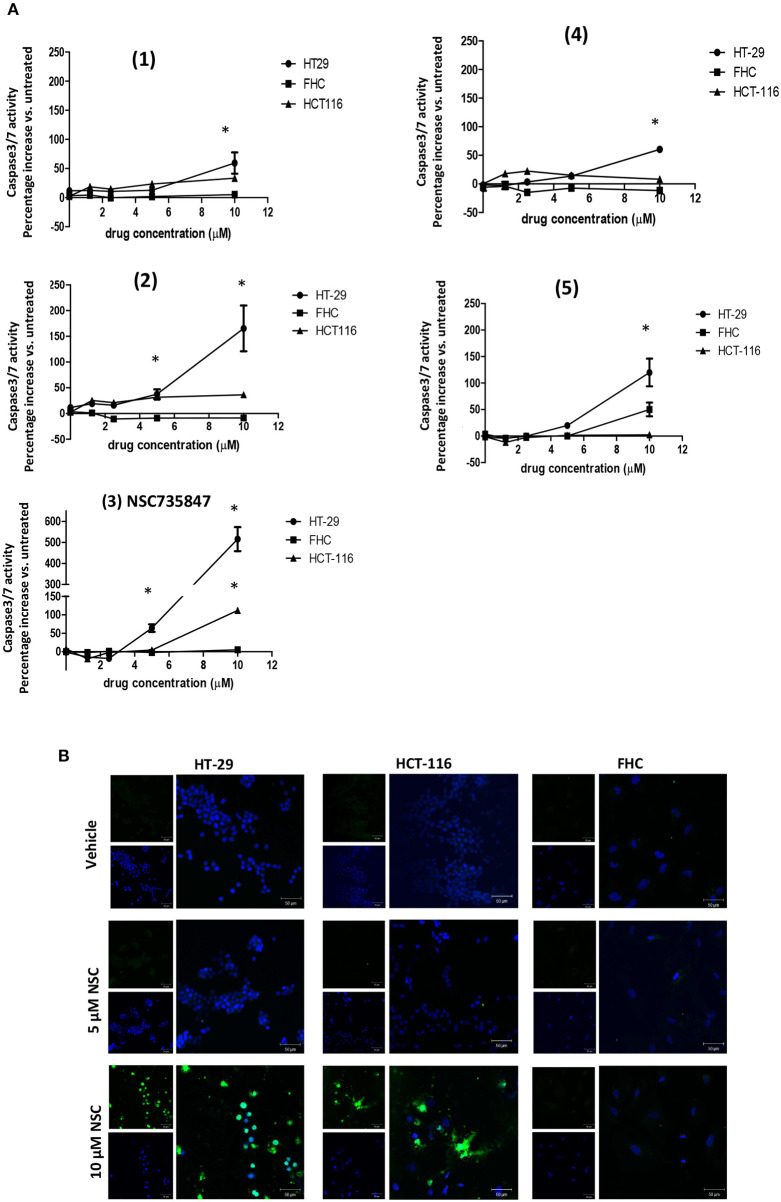
NSC735847 caused apoptosis preferentially in human colorectal cancer cells. **(A)** Human colorectal cancer cells (HT29 and HCT-116) and non-tumorigenic colon cells (FHC) were treated for 16 h with vehicle (0.1% DMSO) or the dihydroartemisinin dimers at doses of 1.25, 2.5, 5, and 10 μM. Caspase-3/7 activity was measured by utilizing the Caspase-Glo 3/7 reagent according to the manufacturer's instructions. Data are represented as the mean ± SEM (*indicates a statistically significant difference between tumor and non-tumor cells, *P* < 0.05). **(B)** HT29, HCT-116 and FHC cells were treated with 5 μM of NSC735847 (NSC), 10 μM of NSC735847, or vehicle (0.1% DMSO) for 16 h. DNA fragmentation was measured by performing TUNEL assays. TUNEL positive cells (green fluorescence) and nuclear DAPI staining (blue fluorescence) were detected by confocal microscopy.

To investigate whether the DHA dimers caused cell death via apoptosis, caspase 3/7 activity was measured. NSC735847 significantly increased caspase 3/7 activity in HT-29 and HCT-116 but not in FHC cells (Figure 2A). Consistent with these results, NSC735847 increased the quantity of TUNEL positive cells in both CRC cell lines however, TUNEL positive cells were absent in NSC735847-treated FHC cells (Figure 2B). Similar to the cytotoxicity study, compounds **(4)** and **(5)** preferentially induced apoptosis in HT-29 compared to FHC cells (Figure 2A) and compound **(1)** did not exhibit tumor selectivity. Interestingly, although compound **(2)** did not display selectivity in the cytotoxicity assays (Figure 1B), the data show an increase in apoptosis in HT29 compared to HCT-116 and FHC cells indicating that this compound elicits death by different mechanisms (e.g., ferroptosis, autophagy, necrosis) in the tested cell lines. Collectively, these results demonstrate that NSC735847 displays superior efficacy and selectivity compared to the structurally related analogs. As such, we investigated the mechanisms of NSC35847 cytotoxicity in our subsequent studies.

### NSC735847-Induced ER Stress Is Required for Apoptotic Cell Death

**Figure 3:**
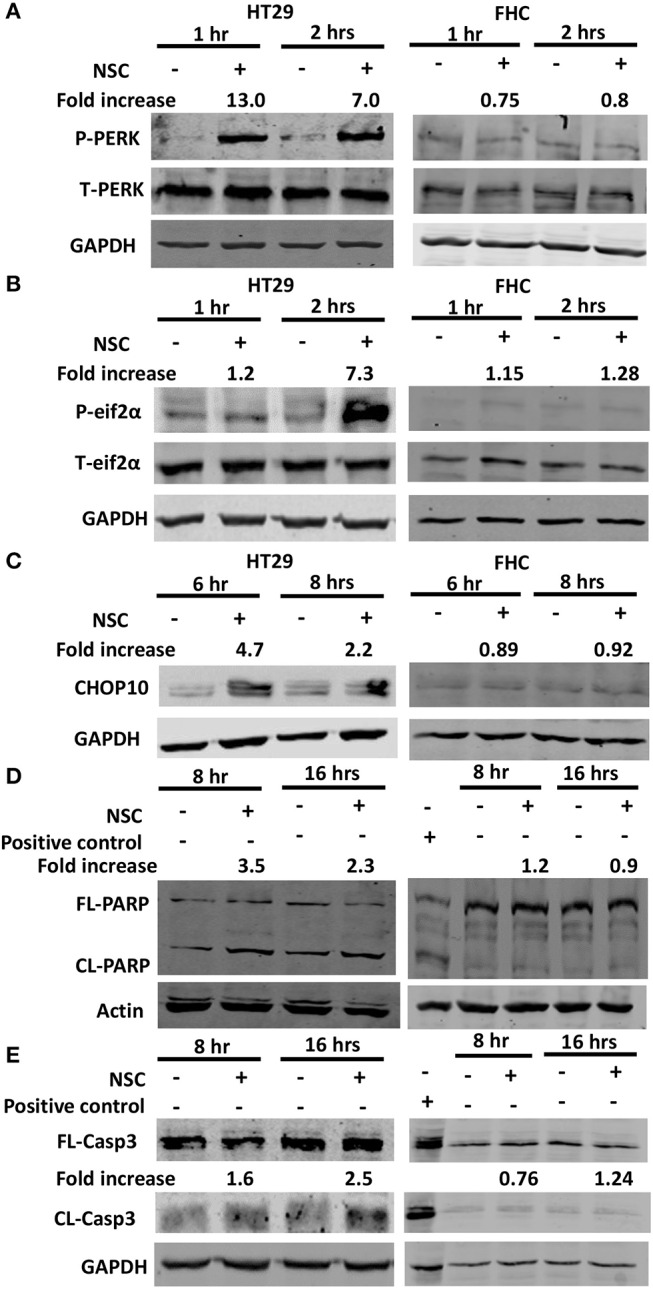
NSC735847 causes ER stress-associated apoptosis selectively in HT29 cells. HT29 and FHC cells were treated with vehicle (0.1% DMSO) or 10 μM NSC735847 (NSC) for 1, 2, 6, 8, and 16 h. Western blot analysis was conducted to examine the levels of **(A)** phospho-PERK (P-PERK) and total PERK (T-PERK) after 1 and 2 h, **(B)** phospho-eIF2α (P-eIF2α) and total eIF2α (T-eIF2α) after 1 and 2 h, **(C)** CHOP10 after 6 and 8 h, **(D)** full length (FL) and cleaved (CL) PARP after 8 and 16 h, and **(E)** FL and CL caspase-3 (Casp3) after 8 and 16 h. Protein band intensities were quantified by using ImageJ software. The fold increase in protein levels were determined by comparing the band intensities of the samples to vehicle-treated cells after normalizing the GAPDH levels. Cell lysates from Jurkat cells that were treated with 25 μM etoposide (Cell Signaling Technologies) were used as a positive control for apoptotic cells.

In response to ER stress, sensors including IRE1 and ATF6, and PERK are activated. PERK undergoes autophosphorylation and then it phosphorylates eIF2a, which causes a global blockade in protein synthesis to allow ER homeostasis to be reestablished (23). However, severe ER stress leads to a substantial increase in the expression of CHOP10, a protein that transactivates pro-apoptotic genes (24). Our previous studies using different cancer cell lines, showed that NSC735847 increased the expression of PERK and BiP, two ER stress regulatory proteins (18). Therefore, the current study investigated whether ER stress was required for NSC735847-induced cytotoxicity. In NSC735847 treated HT-29 cells, the phosphorylation of PERK and its substrate, eIF2α, were increased by more than 7-fold compared to vehicle-treated cells (Figure 3A). To determine if NSC735847 also activated the apoptotic ER stress pathway, we examined the expression of CHOP10 as well as the activation of caspase-3 and PARP. NSC735847 caused a marked increase in CHOP10 expression (Figure 3C) and it stimulated the cleavage of both PARP (Figure 3D) and caspase-3 (Figure 3E). Interestingly, NSC735847 did not increase the activation of PERK, eIF2a, CHOP10, PARP, or caspase 3 in non-tumorigenic, FHC colon cells (Figures 3A–E).

**Figure 4:**
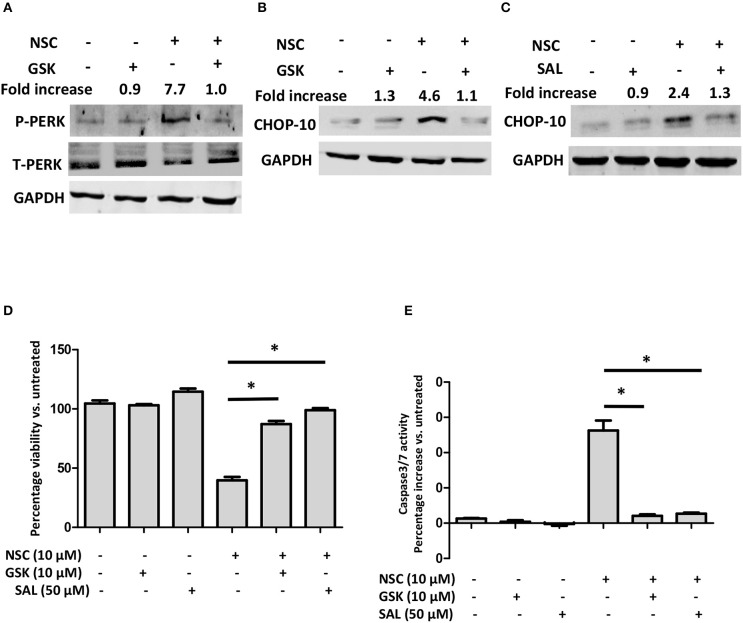
ER stress is required for NSC735847-induced apoptosis in HT29 cells. **(A,B)** HT29 cells were pretreated with 10 μM GSK2606414 (GSK) or vehicle (0.05% DMSO) for 1 h and then the cells were treated with 10 μM of NSC or vehicle (0.05% DMSO). The protein levels of **(A)** phospho-PERK (P-PERK) and total PERK (T-PERK) and **(B)** CHOP10 were detected after 1 h and 8 h, respectively. **(C)** HT29 cells were pretreated with 50 μM salubrinal or vehicle (0.05% DMSO) for 1 h and then treated with 10 μM of NSC or vehicle (0.05% DMSO) for 8 h. The expression of CHOP10 protein was examined by conducting Western blot analysis. Band intensities were quantified by using ImageJ software. The fold increase in protein levels was determined by comparing the band intensities of samples to vehicle-treated cells after normalizing the GAPDH levels. **(D,E)** HT29 cells were pretreated with 10 μM GSK, 50 μM SAL, or vehicle (0.05% DMSO) for 1 h and then the cells were treated with 10 μM of NSC or vehicle (0.05% DMSO). **(D)** Cell survival was measured by conducting MTS experiments after 24 h. **(E)** Caspase-3/7 activity was measured by using Caspase-Glo 3/7 reagent after 16 h of treatment. The data in **(D,E)** are represented as mean ± SEM (*indicates a statistically significant difference between the samples and NSC treated cells, *P* < 0.05).

To evaluate whether ER stress was required for NSC735847 induced apoptosis, the ER stress pathway was blocked using the pharmacological ER stress inhibitors, GSK2606414 and salubrinal. GSK2606414 is a selective, small-molecule inhibitor of PERK that binds to its active site and inhibits ER stress induced PERK autophosphorylation (38). Pretreatment of HT29 cells with GSK2606414 decreased the phosphorylation of PERK (Figure 4A) and the expression of CHOP10 (Figure 4B). In addition, GSK2606414 suppressed NSC735847-mediated cell death (Figure 4D) and inhibited caspase 3/7 activity (Figure 4E). These findings were confirmed with salubrinal, a small-molecule inhibitor of PP1/GADD34 phosphatase activity. Salubrinal inhibits the dephosphorylation of P-eIF2α thereby promoting sustained blockade of mRNA translation which prevents ER stress from occurring (39). Consistent with the data that were obtained with GSK2626414, salubrinal blocked CHOP10 expression (Figure 4C), cell death (Figure 4D), and apoptosis (Figure 4E). Hence, the ER stress pathway is essential for the cytotoxic activity of NSC735847.

### NSC735847 Induces Cell Death in a Heme-Dependent but Iron-Independent Manner

**Figure 5:**
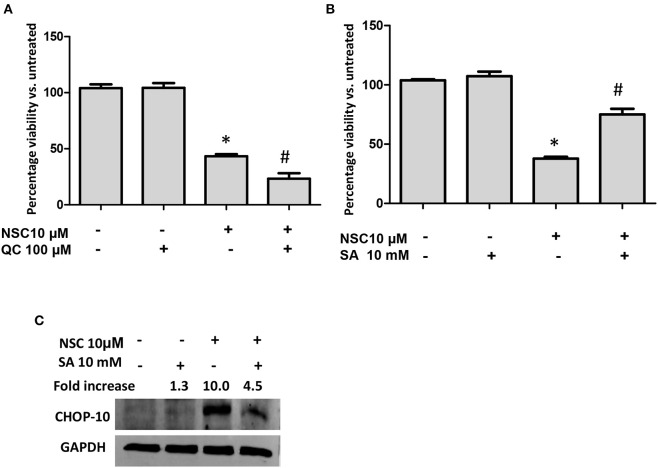
Heme is essential for NSC735847-induced ER stress and apoptosis. HT29 cells were pretreated for 2 h with **(A)** the heme oxygenase inhibitor QC-308 (100 μM), **(B)** the heme biosynthesis inhibitor succinylacetone (SA, 10 mM), or vehicle (0.05% DMSO) and then the cells were treated for 24 h with 10 μM NSC or vehicle (0.05% DMSO). Cell viability was measured by performing MTS assays. **(C)** HT29 cells were pretreated for 2 h with SA (10 mM) or vehicle (0.05% DMSO) and then the cells were treated for 8 h with 10 μM NSC or vehicle (0.05% DMSO). The expression of CHOP10 protein was examined by conducting Western blot analysis. The fold increase in CHOP10 was determined by using ImageJ software after normalizing the GAPDH levels. Data are represented as the mean ± SEM (*indicates a statistically significant difference between the samples and vehicle-treated cells, *P* < 0.05, ^#^indicates a statistically significant difference between the samples and NSC735847-treated cells, P < 0.05).

Several studies have determined that heme is required to catalyze the cleavage and subsequent activation of the endoperoxide bridge in artemisinin-type molecules (40–43). We also demonstrated that heme was needed for the cytotoxicity of NSC735847 in HL-60 and PC-3 cells (18). Therefore, the role of heme in NSC735847-treated colon cancer cells was examined by pretreating the cells with the heme oxygenase inhibitor, QC-308, or the heme biosynthesis inhibitor, succinylacetone. QC-308 is a selective inhibitor of heme oxygenase-1 (HO-1), the primary enzyme that is responsible for catabolism of heme to biliverdin, carbon monoxide, and ferrous iron (44). On the other hand, succinylacetone is an inhibitor of aminolevulinic acid dehydratase, a key enzyme in heme synthesis (45). Blockade of HO-1 activity with QC-308 increased the cytotoxicity of NSC735847 (Figure 5A). In line with this finding, the inhibition of heme synthesis with succinylacetone rescued the cells from the cytotoxic effects of NSC735847 (Figure 5B). Since our results showed that heme was essential for the anticancer activity of NSC735847, and heme has been reported to induce ER stress (46), we examined whether preventing heme synthesis with succinylacetone suppressed NSC735847-induced ER stress. As shown in Figure 5C, the inhibition of heme synthesis with succinylacetone blunted NSC735847-induced CHOP10 expression. These results indicate that heme is necessary for ER stress-induced death in NSC735847-treated cells.

**Figure 6:**
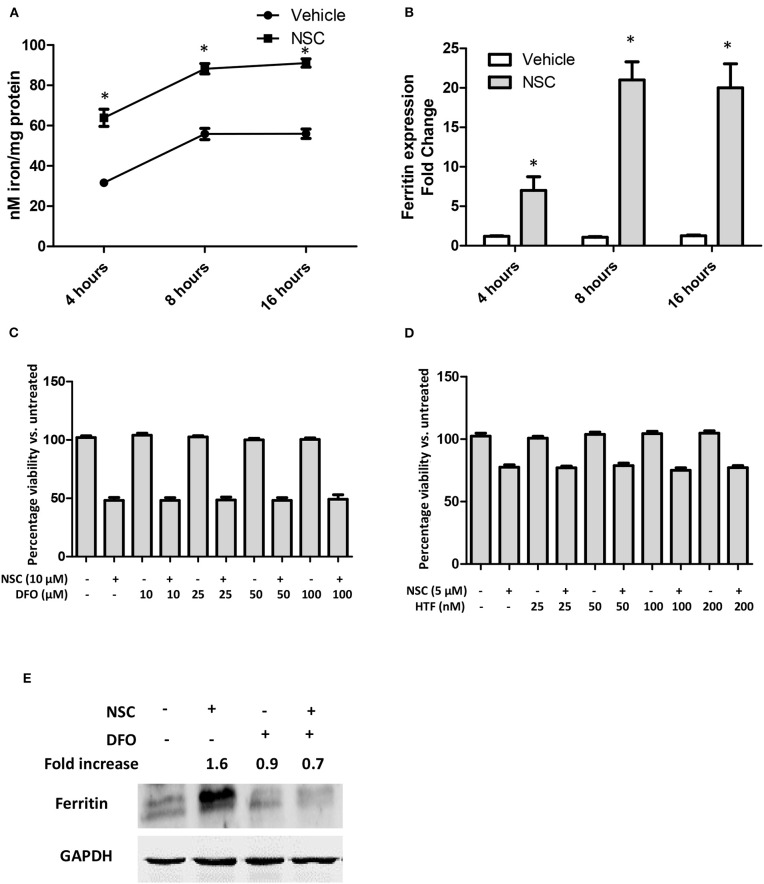
NSC735847 induces cell death in an iron-independent manner in HT29 cells. **(A,B)** HT29 cells were treated with 10 μM NSC or vehicle (0.1% DMSO) for 4, 8, and 16 h. **(A)** Intracellular iron levels were measured by using the iron Enzyme Linked Immunosorbent Assay (ELISA) QuantiChrom kit as instructed by the manufacturer. **(B)** The levels of ferritin mRNA were measured by conducting quantitative real-time PCR (qRT-PCR). The fold increase in ferritin transcripts was calculated using the method described by (37). **(C)** HT29 cells were pretreated for 4 h with different concentrations (10, 25, 50, and 100 μM) of deferoxamine (DFO) or vehicle (0.05% DMSO) and then the cells were treated for 24 h with 10 μM of NSC or vehicle (0.05% DMSO). Cell survival was measured by conducting MTS viability assays. **(D)** HT29 cells were pretreated with different concentrations (25, 50, 100, and 200 nM) of holo-transferrin (HTF) or vehicle (0.05% DMSO) for 2 h and then the cells were treated for 24 h with 5 μM NSC or vehicle (0.05% DMSO). Cell survival was measured by performing MTS cell viability assays. Data in **(A–D)** are represented as the mean ± SEM (*P* < 0.05). **(E)** HT29 cells were pretreated with 100 μM DFO or vehicle (0.05% DMSO) for 4 h then the cells were treated for 24 h with 10 μM of NSC or vehicle (0.05% DMSO). The levels of ferritin protein were examined by performing Western blot analysis. The fold increase in ferritin protein was determined by using ImageJ software after normalization of the GAPDH levels. *Indicates a statistically significant difference between the samples and vehicle-treated cells.

The heme catabolic product, iron, is also important for the activity of artemisinin molecules (47, 48). Hence, we evaluated the impact of NSC735847 on intracellular iron levels. Cell treatment with NSC735847 caused a time-dependent increase in cellular free iron (Figure 6A). It is well-established that elevated levels of free iron cause cellular toxicity by forming ROS via Fenton reactions. To prevent this ROS-induced cytotoxicity, cells increase the expression of the iron storage protein, ferritin. Consistent with the increase in free iron, NSC735847 upregulated the expression of ferritin mRNA (Figure 6B). To determine the requirement for iron in the cytotoxicity of NSC735847, HT-29 cells treated with deferoxamine (DFO), a selective chelator that reduces intracellular iron levels (49–51). To our surprise, DFO did not prevent NSC735847-mediated cell death (Figure 6C). To confirm that the cellular iron levels were sufficiently reduced by DFO, we measured ferritin protein expression as a surrogate marker of iron levels. DFO inhibited NSC735847-mediated ferritin expression indicating that minimal quantities of free iron were present in the cells (Figure 6E). To provide additional support for this finding, iron was supplied to the cells by pretreating them with the iron-loaded transferrin molecule, holo-transferrin (HTF) (52–54). HTF did not enhance the killing effect of NSC735847, verifying that iron was dispensable for NSC735847-induced cytotoxicity (Figure 6D).

### Oxidative Stress Is Not Essential for the Cytotoxic Action of NSC735847 in Colon Cancer Cells

**Figure 7:**
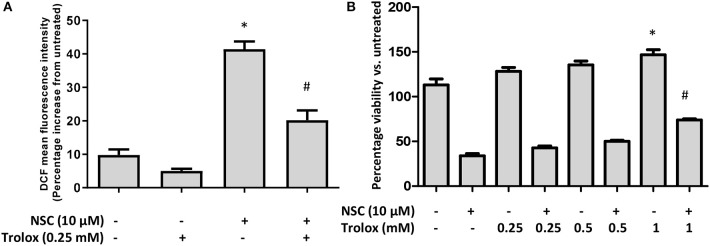
Oxidative stress is not necessary for NSC735847-induced cell death in HT29 cells. **(A)** HT29 cells were pretreated with 0.25 mM Trolox or vehicle (0.05% DMSO) for 2 h and then treated for 8 h with 10 μM NSC or vehicle (0.05% DMSO). Oxidative stress was measured by conducting CM-H_2_DCFDA microplate assays. Fluorescence was measured and is presented as a percent increase compared to untreated cells. **(B)** HT29 cells were pretreated for 2 h with different concentrations of Trolox (0.25, 0.5, and 1 mM) or vehicle (0.05% DMSO) and then the cells were treated for 24 h with 10 μM of NSC or vehicle. Cell survival was measured by performing MTS cell viability assays. Data are represented as the mean ± SEM (*indicates a statistically significant difference between the samples and vehicle treated cells, ^#^indicates a statistically significant difference between the samples and NSC treated cells, *P* < 0.05).

Several studies have reported that artemisinin-induced cell death is driven by the oxidative stress pathway (55). We also showed that NSC735847 increased oxidative stress in the promyelocytic leukemia cell line, HL-60 and the human prostate cancer cell line, PC3 (18). Therefore, the effect of oxidative stress on the cytotoxicity of NSC735847 in HT-29 cells was investigated by utilizing the oxidative stress probe, CM-H_2_DCFDA (DCF). NSC735847 increased the intracellular oxidative stress levels as indicated by the increase in DCF fluorescence (Figure 7A). To investigate whether oxidative stress was a requirement for cell death the antioxidant, Trolox, was employed. Trolox is a water-soluble analog of vitamin E that exhibits antioxidant activity by scavenging free radicals and prohibiting lipid peroxidation (56, 57). In HT29 cells that were pretreated with Trolox, and then treated with NSC735847, a significant reduction in oxidative stress occurred (Figure 7A). However, blockade of oxidative stress with 0.25 or 0.5 mM Trolox did not rescue the cells from NSC735847-induced cytotoxicity (Figure 7B). Although 1 mM Trolox significantly increased cell survival in NSC735847-treated cells, a similar increase occurred in cells treated with 1 mM Trolox alone, indicating that the enhanced survival was caused by Trolox rather than the inhibition of NSC7358457-induced ROS (Figure 7B). Collectively, this data indicates oxidative stress is not required for the antitumor activity of NSC735847.

### NSC735847 Increases the Cytotoxicity of Clinical CRC Therapeutics in Colon Cancer Cells

**Figure 8:**
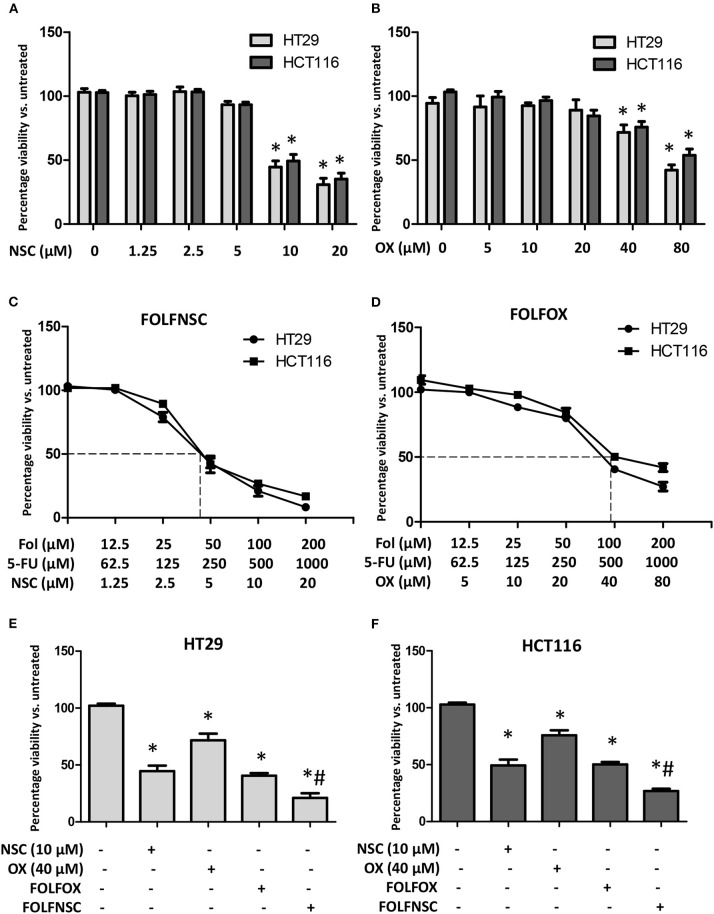
The combination of NSC735847, 5-Fluorouracil, and folinic acid is more cytotoxic than FOLFOX in human CRC cells. **(A,B)** HT29 and HCT116 cells were treated for 24 h with vehicle (0.1% DMSO) or different concentrations of **(A)** NSC735847 (NSC) (1.25, 2.5, 5, 10, and 20 μM) or **(B)** oxaliplatin (OX) (5, 10, 20, 40, and 80 μM) and the viability of the cells was determined by conducting MTS experiments. **(C,D)** HT29 and HCT116 cells were treated for 24 h with vehicle or different concentrations of **(C)** NSC, 5-Fluorouracil (5-FU), and folinic acid (Fol) (FolFNSC) or **(D)** 5-FU, folinic acid, and oxaliplatin (FOLFOX) and then cell survival was determined by conducting MTS viability assays. **(E)** HT29 and **(F)** HCT116 cells were treated for 24 h with 10 μM NSC, 40 μM OXA, FOLFOX (500 μM 5-FU, 100 μM Fol, 40 μM OX), or FolFNSC (500 μM 5-FU, 100 μM Fol, 10 μM NSC) and cell survival was measured by performing MTS viability assays. The data are represented as the mean ± SEM (*indicates a statistically significant difference between the samples and vehicle treated cells, ^#^indicates a statistically significant difference between the samples and FOLFOX treated cells, *P* < 0.05).

Most cancer chemotherapeutic regimens for advanced CRC employ multiple drugs (58). In this approach, the use of low-dose agents with different mechanisms of action is designed to increase drug activity, reduce drug resistance, and decrease overall adverse effects. One such combination, FolFOX, is composed of folinic acid (Fol; also known as leucovorin), 5-fluorouracil (F or 5-FU), and oxaliplatin (OX) and it is the standard of care treatment for advanced CRC (59). However, the OX in FolFOX often causes severe, grade 3 neurotoxicities (60) which can limit its utility. Therefore, drugs that have distinct mechanisms of action from 5-FU and Fol and exhibit selective toxicity are needed to improve the efficacy and safety of CRC therapy. Artemisinin-type drugs are well-tolerated and these agents have been reported to enhance the activity of FDA-approved antineoplastic agents including doxorubicin and gemcitabine (61). Moreover, our data show that NSC735847 is selectively toxic toward CRC cell lines (Figures 1, 2). Hence, the antitumor effect of co-administered 5-FU, Fol, and NSC735847 (FolFNSC) was examined. When administered as single agents, both NSC735847 and OX induced death in a concentration-dependent manner in HT29 and HCT116 cells although NSC735847 was lethal at substantially lower concentrations (Figures 8A,B). Next, the cytotoxic activity of FolFNSC was compared to FolFOX and at the tested concentrations, FolFNSC reduced the survival of HT29 and HCT116 cells at a lower concentration than FolFOX (Figures 8C–F). These results show that FolFNSC is an effective drug combination that exhibits greater cytotoxicity than FolFOX in colorectal cancer cells. As such, FolFNSC may be an appropriate regimen for FolFOX patients with severe OX-mediated toxicities.

## Discussion

**Figure 9:**
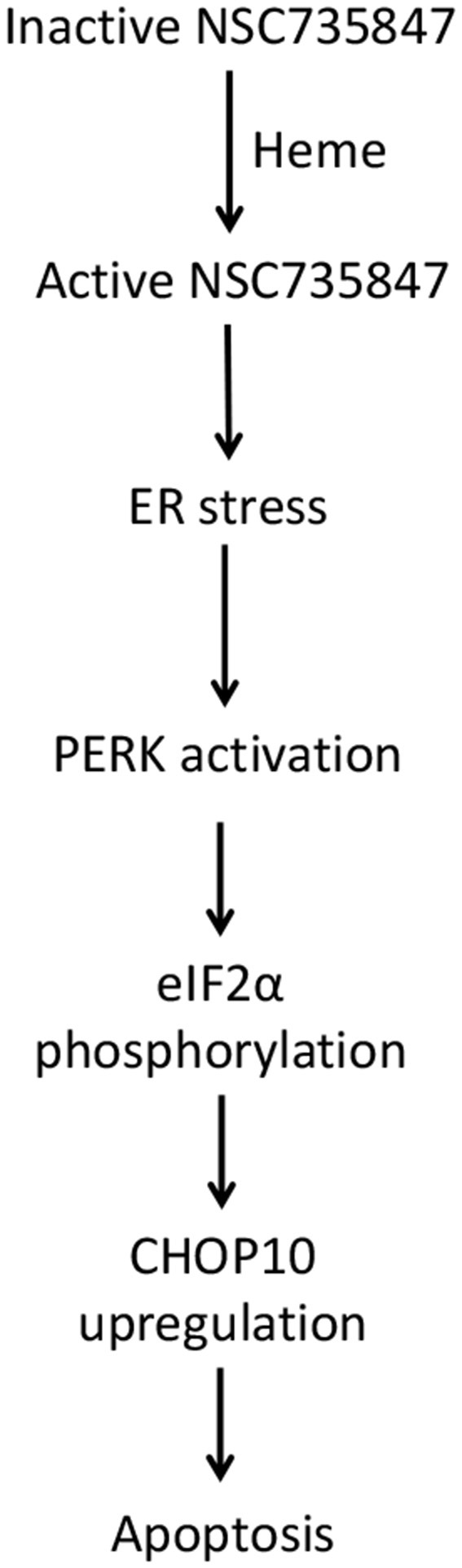
Proposed mechanism of NSC735847 cytotoxicity in colorectal cancer cells. NSC735847 is bioactivated by heme. The active drug then activates PERK, eIF2α, CHOP10, and apoptosis.

Colorectal cancer (CRC) causes significant morbidity and mortality throughout the world. CRC rates are increasing in young adults at an alarming rate which may be due to lifestyle and genetic factors (62). Hence, new therapeutic agents are urgently needed to improve outcomes for the increasing number of individuals who will be diagnosed with CRC. In this study, the anticancer activity of NSC735847 and its derivatives was examined to identify a lead compound that displayed efficacy and selectivity toward CRC cells. It was determined that NSC735847 was more lethal against CRC cells than the derivatives of NSC735847. In addition, NSC735847 was cytotoxic toward tumorigenic but not non-tumorigenic colon cells. Our data suggests that NSC735847 is bioactivated by heme and the activated molecule triggers ER stress mediated apoptosis (Figure 9). Although, oxidative stress and iron were increased in NSC735847-treated cells these molecules were not needed to elicit cell death. The data also showed that FolFNSC was capable of inducing colon tumor cell death at lower concentrations than FolFOX. Collectively, these findings suggest that NSC735847 may be a safe and effective component of combination therapy for CRC.

Although CRC therapy is continually improving, it has been a challenge to identify effective regimens that produce minimal adverse effects. These adverse events are commonly caused by drug activity in off-target tissues or sites. Therefore, agents that are more toxic toward their target (i.e., cancer cells) than to non-tumor host cells exhibit selective toxicity and allow the patient to better tolerate the drug. It has been proposed that ER stress inducing agents elicit selective toxicity by exploiting the fact that endogenous ER stress levels are higher in cancer than in non-cancer cells. Under these conditions, ER stress inducers cause cancer cells to reach the death threshold more readily than non-cancer cells. ER stress mediated selective toxicity has been demonstrated in numerous studies. For example, cell death occurred in A549 lung cancer cells that were transfected with the ER stress inducing transcript, melanoma differentiation associated gene-7 (mda-7, also known as IL-24), however mda-7 did not affect the survival of non-tumor bronchial epithelial cells (63, 64). In addition, HA15, a small molecule that activates ER stress by inhibiting the interaction between BIP and ER stress sensors (PERK, IRE1, and ATF6), was lethal to melanoma but not non-tumorigenic melanocytes (65). Moreover, our previous study showed that the prostamide, 15d-PMJ_2_, induced ER stress dependent death in melanoma and non-melanoma skin cancer cells but not in non-tumorigenic melanocytes or keratinocytes (66). In the current study, NSC735847 induced greater ER stress and death in tumorigenic compared to non-tumorigenic colonocytes. We also observed that ER stress was required for tumor cell death. These findings indicate that the ER stress pathway drives the selective action of NSC735847. This tumor selectivity may also explain why NSC735847 generated negligible toxicity in our previous *in vivo* study (29). Specifically, NSC735847 and NSC735847 + topotecan did not cause significant changes in the body weight of athymic nude mice that bore subcutaneous or orthotopic non-small cell lung cancer lesions, indicating the absence of overt toxicity. Moreover, the tumor volume, tumor weight/body weight ratio, and animal mortality were substantially reduced in mice treated with NSC735847 + topotecan compared to untreated animals, demonstrating the efficacy of NSC735847. Since numerous artemisinins and ER stress inducers are being tested clinically (NCT01132911, NCT03529448, NCT00764036, NCT03100045), it is probable that other agents within this class will display efficacy and minimal toxicity as these pre-clinical properties must be demonstrated before agents advance to clinical trial. With this information in mind, it is tempting to speculate that FolFNSC will be an effective agent with a favorable safety profile in pre-clinical and clinical CRC.

The bioactivation of DHA-type molecules has been reported to be mediated by iron- and/or heme-induced cleavage of its endoperoxide bridge (18, 41, 47, 48, 67). Once activated, DHAs become reactive molecules that generate ROS which interacts with DNA, proteins, and lipids to produce lethal cell damage (42, 68). As such, we investigated the role of ROS, iron, and heme in the antitumor activity of NSC735847. Three important findings led us to conclude that iron and ROS were not required for the lethality of NSC735847. First, iron depletion did not suppress the cytotoxicity of NSC735847. Second, providing supplemental iron through the use of holotransferrin did not increase the cytotoxicity of NSC735847. Third, NSC735847 caused ROS production but neutralizing the ROS with an antioxidant did not inhibit NSC735847 mediated cell death. The finding that ROS was not essential for the activity of NSC735847 in colon cancer cells was unexpected because our previous data demonstrated that NSC735847-generated ROS reduced the viability of promyelocytic leukemia and prostate cancer cell lines (18). However, other groups have demonstrated that artemisinin-like molecules initiate death independent of ROS in leukemia, hepatoma, and breast cancer cells (49, 69), therefore the requirement for ROS appears to be cell type dependent.

Although we found that ROS and iron were non-essential, the presence of heme was a prerequisite for the antitumor effect of NSC735847. Specifically, ER stress induced death was suppressed in cells that were pretreated with a heme synthesis inhibitor. Moreover, NSC735847 mediated death was augmented in colon cancer cells whose heme levels were stabilized by inhibiting heme degradation. Similar to our findings, Zhang et al., showed that heme, but not iron, was a critical driver of the antitumor activity of artemisinins (42). Collectively, these data suggest that heme activated NSC735847 induces colon tumor cell death via the ER stress pathway.

The co-administration of agents that have different mechanisms of action is an effective approach for decreasing drug resistance and increasing drug efficacy. By combining agents with distinct actions, different pro-tumorigenic signaling pathways are targeted simultaneously (70, 71). FolFOX, a three-drug combination that includes 5-FU, folinic acid, and oxaliplatin, is the standard of care regimen for CRC (59). 5-FU is an antimetabolite that inhibits the synthesis of both RNA and DNA and it also blocks the activity of thymidylate synthase (TS). Folinic acid is an additive that increases the affinity of 5-FU for TS while oxaliplatin alkylates DNA. The studies herein determined that NSC735847 is a heme-dependent, inducer of ER stress apoptosis. Since this mechanism of cytotoxicity is distinct from that of Fol and 5-FU, a goal of this study was to examine the antitumor activity of FolFNSC to determine if NSC735847 could be a suitable alternative for OX in patients who are unable to tolerate its adverse effects. According to our data, NSC735847 was cytotoxic toward CRC cells at lower concentrations than oxaliplatin. Furthermore, when combined with folinic acid and 5-FU, lower concentrations of FolFNSC than the FolFOX were needed for its tumor cell killing effect. These findings are consistent with previous reports which showed that artemisinin-type drugs increased the activity of FDA approved anticancer agents (61). Moreover, in a phase II clinical trial the combination of artesunate (a derivative of artemisinin) and CRC drugs showed promising antineoplastic activity and was well-tolerated (72). Furthermore, since NSC735847 exhibits selective toxicity, it is possible that FolFNSC will produce an improved safety profile compared to FolFOX. Therefore, additional testing should be conducted to determine if NSC735847 with Fol and 5-FU will aid in achieving the ultimate goal of increasing treatment efficacy and decreasing adverse effects.

## Data Availability Statement

All datasets analyzed in this study are available upon request.

## Author Contributions

AE, ES, and RV contributed to the conception and design of the study. AE, ES, and RV wrote and revised the manuscript. WG and ME synthesized the drugs and revised the manuscript. AE, ES, MM, and PM acquired the data. AE, ES, and RV analyzed and interpreted the data. All authors read and approved the final manuscript.

## Conflict of Interest

ME and WG are employed by the company, ElSohly Laboratories, Inc. The remaining authors declare that the research was conducted in the absence of any commercial or financial relationships that could be construed as a potential conflict of interest.
