# Supplementary material for: Heme-Dependent ER Stress Apoptosis: A Mechanism for the Selective Toxicity of the Dihydroartemisinin, NSC735847, in Colorectal Cancer Cells
Source: Front Oncol. 2020 Jun 17;10:965. doi: 10.3389/fonc.2020.00965 (PMC7313430; doi:10.3389/fonc.2020.00965)

Supp 3A

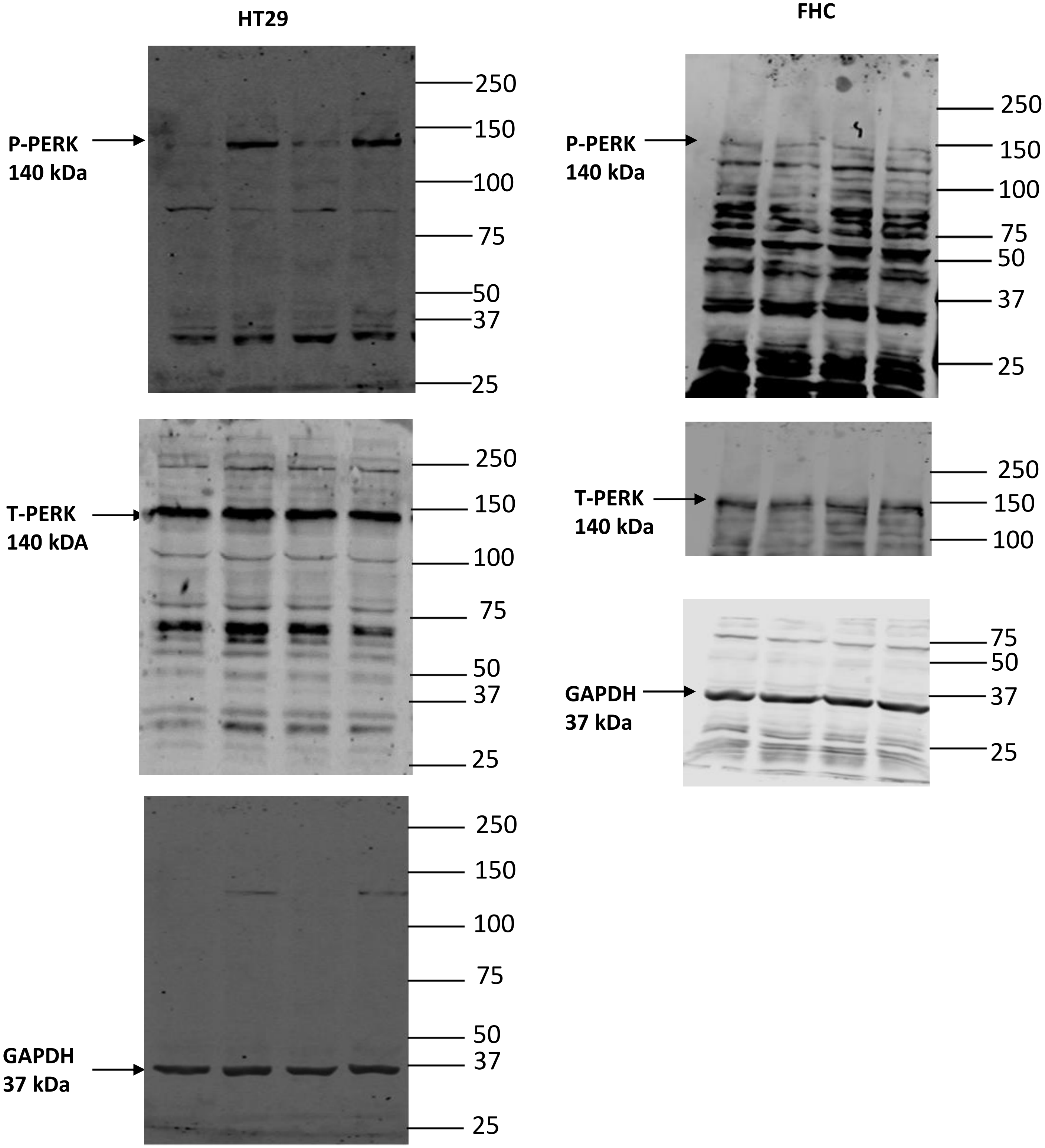
HT29
FHC
P-PERK
140 kDa
T-PERK
140 kDA
GAPDH
37 kDa
P-PERK
140 kDa
T-PERK
140 kDa
GAPDH
37 kDa
250
150
100
75
50
37
25

Supp 3B

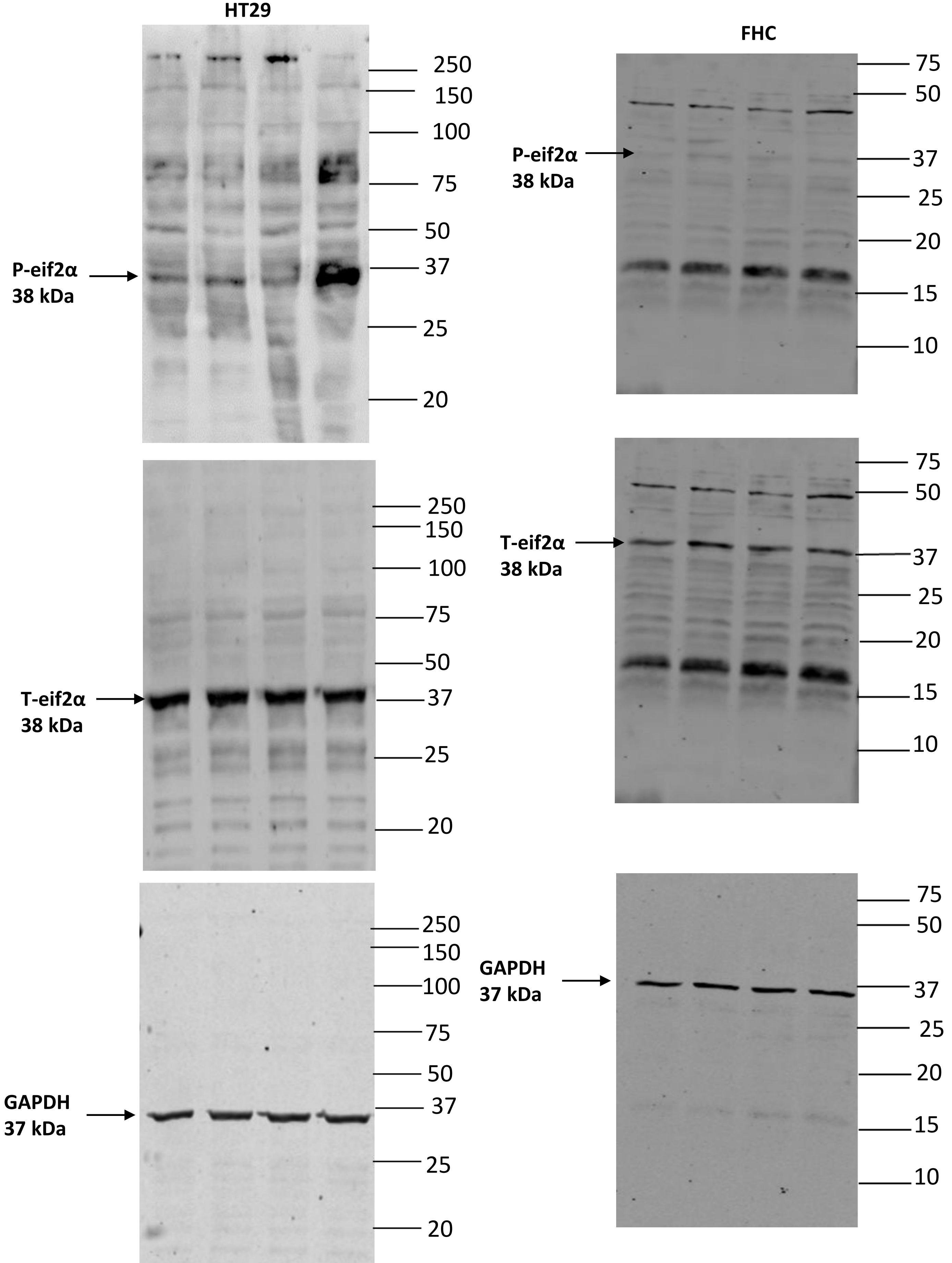
HT29
FHC
P-eif2α 38 kDa
T-eif2α 38 kDa
GAPDH 37 kDa
250
150
100
75
50
37
25
20
15
10

Supp 3C

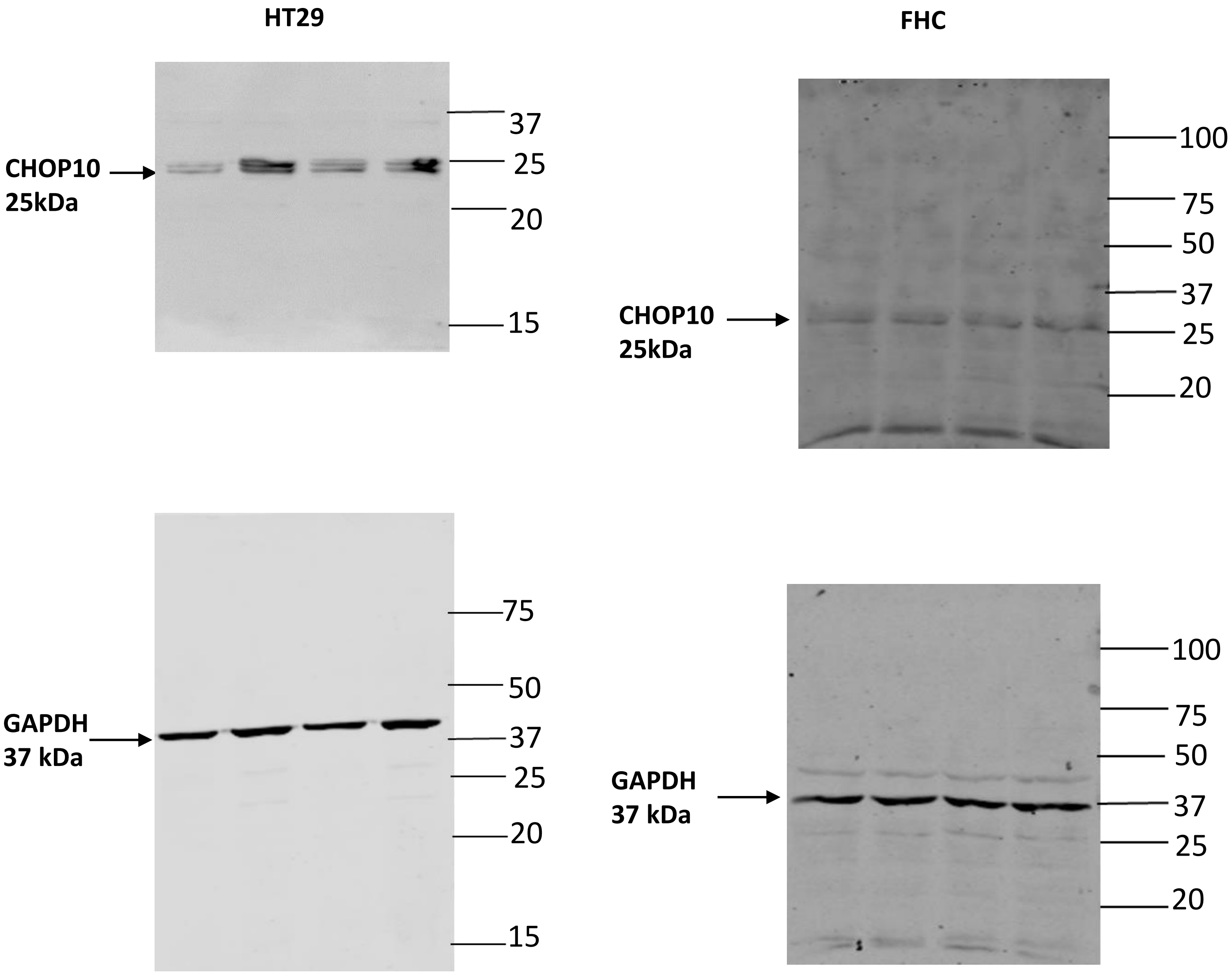
HT29
FHC
CHOP10
25kDa
37
25
20
15
100
75
50
37
25
20
GAPDH
37 kDa
75
50
37
25
20
15
100
75
50
37
25
20

Supp 3D

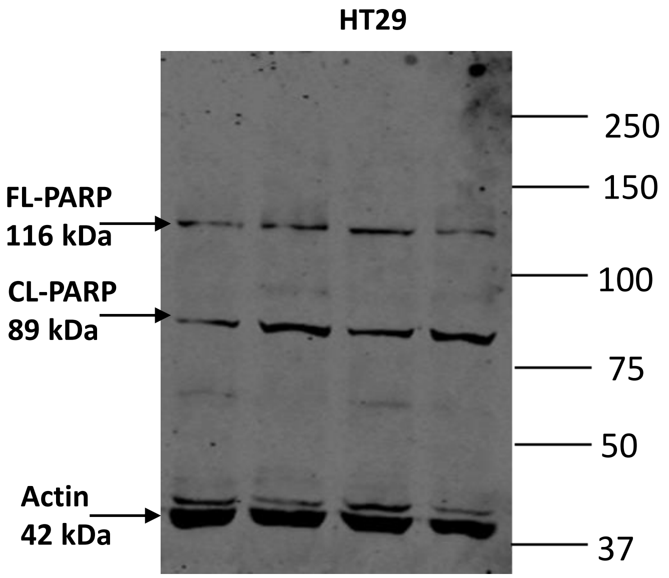

HT29
FL-PARP
116 kDa
CL-PARP
89 kDa
Actin
42 kDa
250
150
100
75
50
37

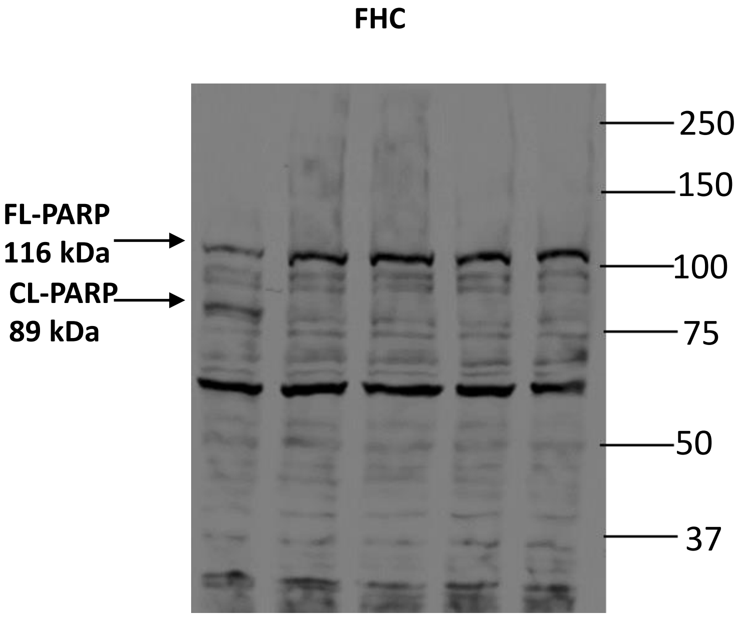

FHC
FL-PARP
116 kDa
CL-PARP
89 kDa
250
150
100
75
50
37

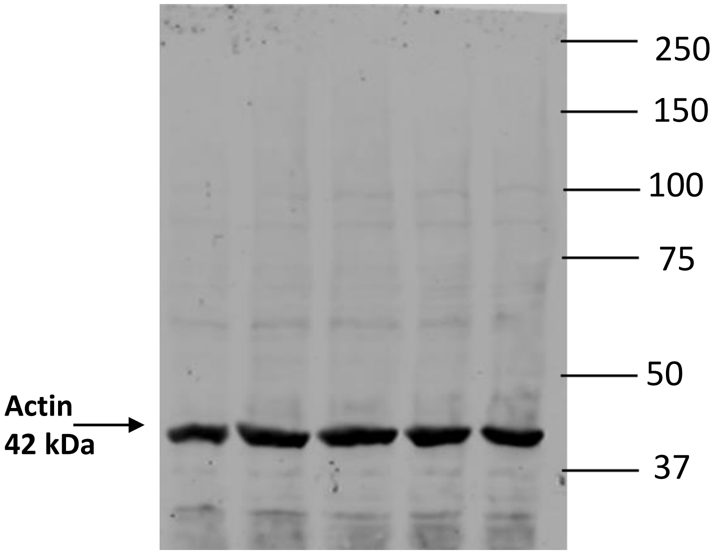

Actin
42 kDa
250
150
100
75
50
37

Supp 3E

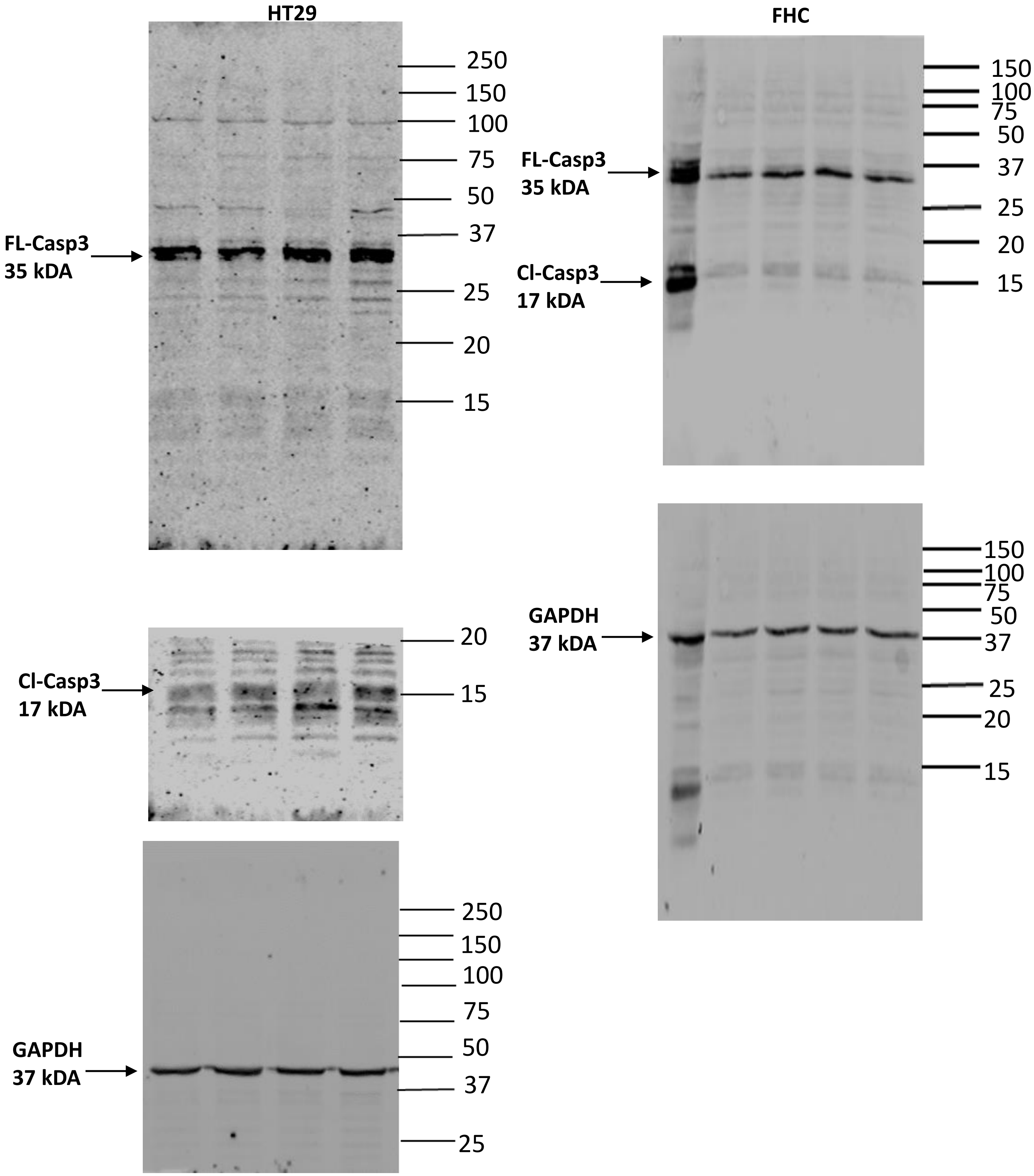
HT29
FL-Casp3
35 kDA
250
150
100
75
50
37
25
20
15
Cl-Casp3
17 kDA
20
15
GAPDH
37 kDA
250
150
100
75
50
37
25
FHC
FL-Casp3
35 kDA
Cl-Casp3
17 kDA
150
100
75
50
37
25
20
15
GAPDH
37 kDA
150
100
75
50
37
25
20
15

Supp 4

4A

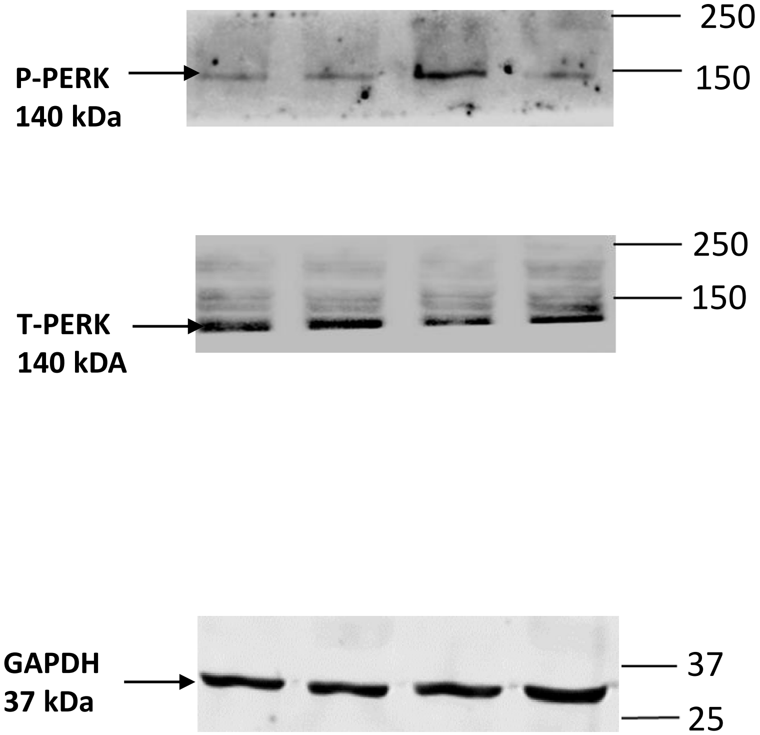

4B

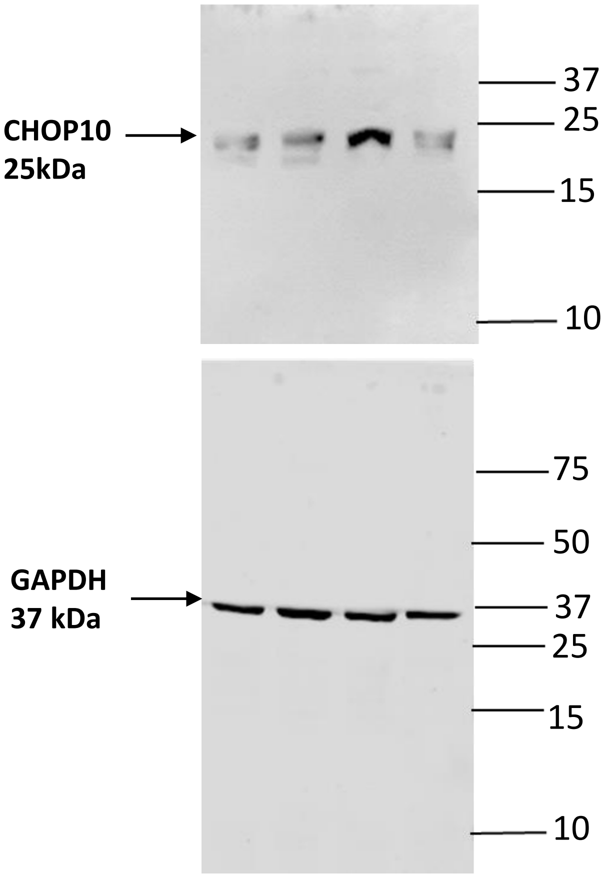

4C

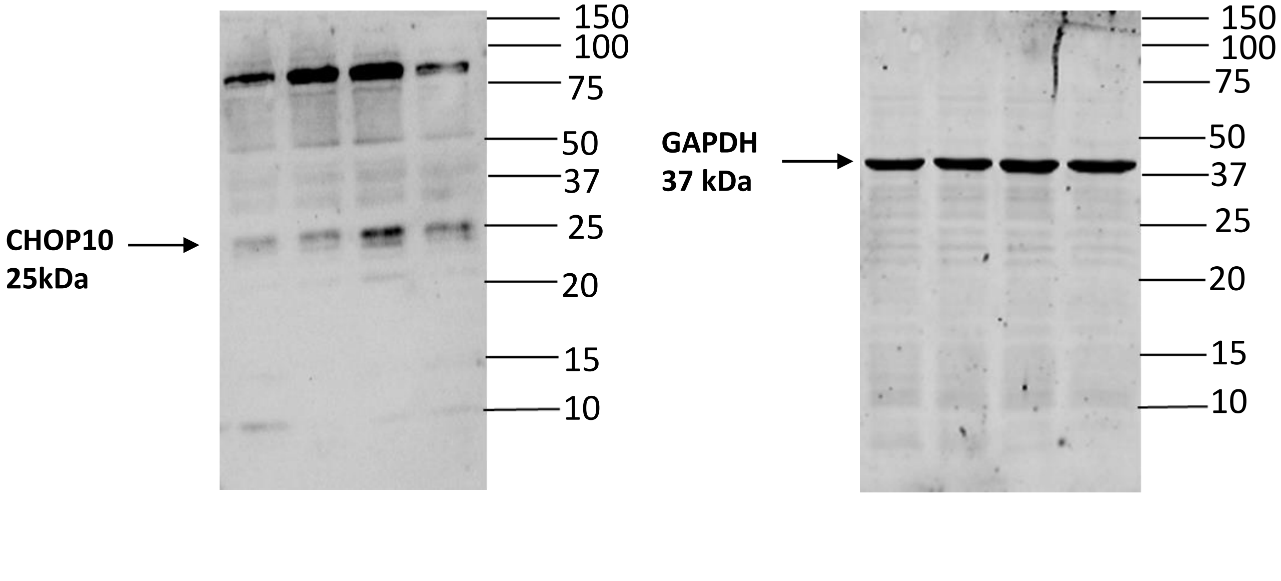

Supp 5C

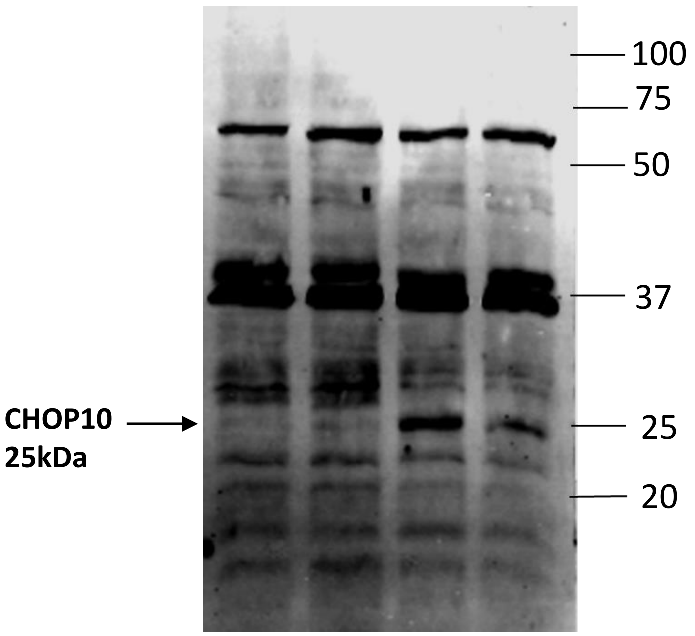

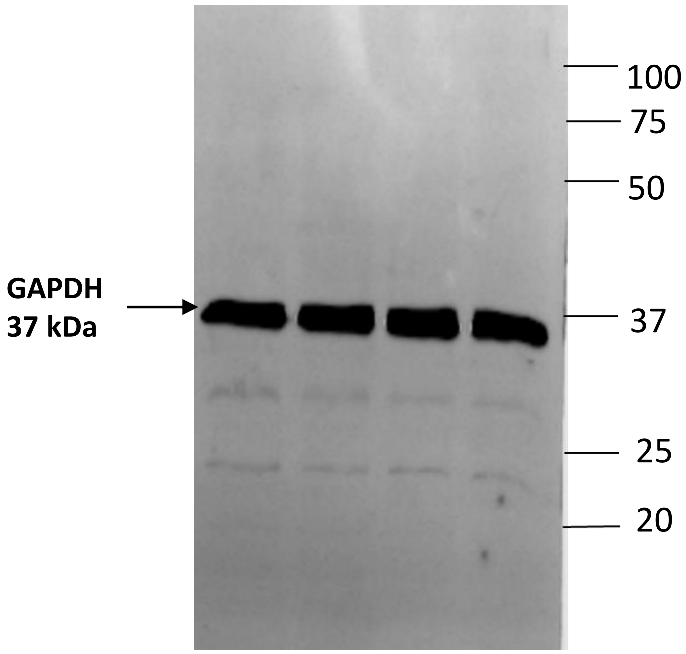

Supp 6E

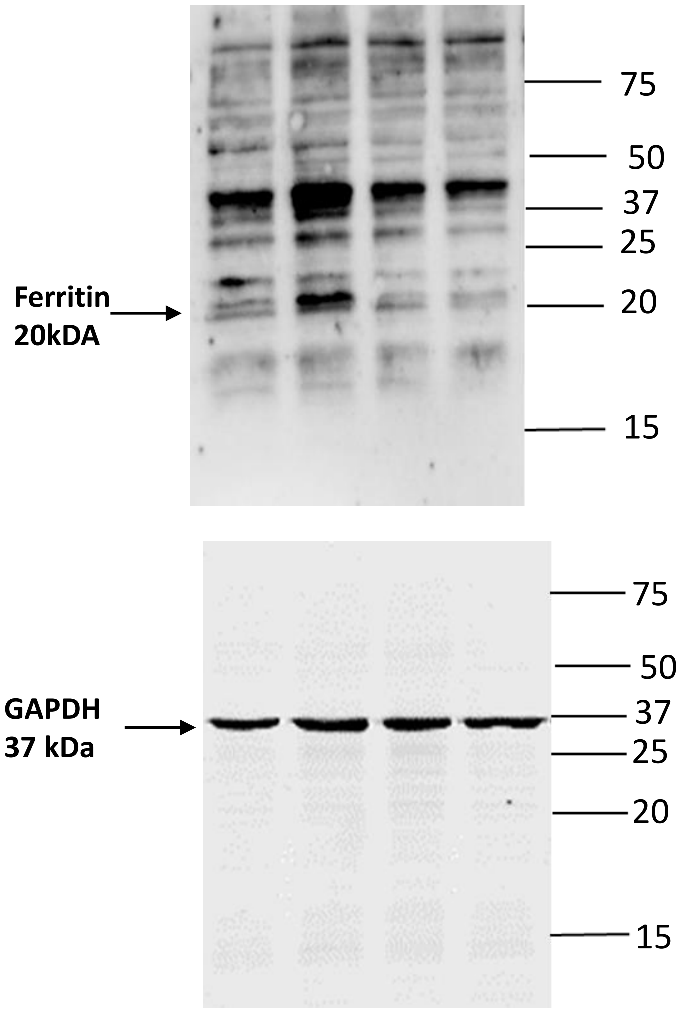

Supplement: Supplementary file 1 [file Data_Sheet_1.pdf]
